# Usefulness of the Reborn Soup for the Reduction of Salt Intake

**DOI:** 10.1155/2024/6090466

**Published:** 2024-08-28

**Authors:** Yuko Ohta, Satoko Sakata, Kazuhiro Ohta, Masahiko Kusano, Ritsuko Fujisawa, Yuji Komorita, Yukie Kuwahara, Yuki Fukamatsu, Hiroshi Tsuruta, Hidetoshi Nakamura, Takuya Tsuchihashi

**Affiliations:** ^1^ Division of General Internal Medicine Kyushu Dental University, Fukuoka, Japan; ^2^ Department of Medicine and Clinical Science Graduate School of Medical Sciences Kyushu University, Fukuoka, Japan; ^3^ Internal Medicine and Gastroenterology Ohta Clinic, Fukuoka, Japan; ^4^ Reborn TK Japan Co. Ltd, Fukuoka, Japan; ^5^ Kokura Daiichi Hospital, Fukuoka, Japan; ^6^ Steel Memorial Yawata Hospital, Fukuoka, Japan

## Abstract

**Aims:**

The purpose of the present study was to investigate the influence of reborn soup on the perceptions of saltiness and palatability.

**Methods:**

Subjects comprised 103 staff working at Kokura Daiichi Hospital (22 males, 81 females, and mean age: 35 ± 12 years old). They tested soups (commercially available soup with 0.9% NaCl solutions (A), commercially available soup with 0.6% NaCl solutions (B), and reborn soup diluted to 0.6% NaCl solutions (C)). Evaluations of saltiness and palatability for each solution were conducted using a visual analog scale in a double-blinded randomized manner. We estimated 24-hour salt excretion using spot urine samples to estimate salt intake and also assessed blood pressure, the awareness of salt intake using a self-description questionnaire score, and other confounding factors including lifestyle factors.

**Results:**

In all subjects, the average estimated salt intake was 9.0 ± 2.0 g/day, and the rates at which subjects met the established salt intake targets were 15.1% in 73 females without hypertension (<6.5 g/day), 23.5% in 17 males without hypertension (<7.5 g/day), and 0.0% in 13 subjects with hypertension (<6.0 g/day). In both saltiness and palatability, B scored significantly lower than A, but C scored significantly higher than B. Salt intake levels were categorized into tertiles (Q1, lowest; Q3, highest). C scored significantly higher for palatability in the Q1 group than in the Q3 group.

**Conclusions:**

Most participants exceeded the established targets of salt intake. The high-salt-intake group might be able to feel less palatable. Our results indicate that reborn soup may be effective in reducing salt intake without loss of palatability.

## 1. Introduction

Excessive salt intake not only raises blood pressure (BP) but also increases the risk of cardiovascular events and cancer [1, 2]. Salt reduction is most important in guidance regarding lifestyle modifications in Japanese hypertensive patients as well as for the health of the general population. Guidelines for the Management of Hypertension by the Japanese Society of Hypertension (JSH 2019) proposed a salt reduction goal for hypertensives of <6 g/day, and the Dietary Reference Intakes for Japanese (DRI-J) 2020 declared set a daily salt intake of <7.5 g/day in men and <6.5 g/day in women as provisional dietary goals [3, 4]. The salt intake of the Japanese population has been gradually decreasing [5]; however, it is still high. We recently reported that the compliance with salt restriction proposed by guidelines is insufficient in Japanese hypertensive patients [6, 7]. Meanwhile, it is well known that limiting the NaCl content in foods can make them less palatable [8]; thus, new approaches are needed to preserve foods' palatability. The addition of an umami substance (monosodium l-glutamate (MSG) or calcium diglutamate (CDG)) has made it possible to lower the NaCl concentration without sacrificing the pleasantness, saltiness, or taste intensity of soups [9–14]. Soup is well known to be a common food consumed not only in Japan but also throughout the world. Therefore, we conducted the present study to investigate the influence of reborn soup on the perceived saltiness and palatability of salt solutions.

## 2. Methods

We recruited subjects who were examined in the morning on their health checkup day (July 2022). There were no participants who withdrew or refused consent for the study. Subjects comprised 103 staff working at Kokura Daiichi Hospital (22 males, 81 females, and mean age: 35 ± 12 years old). All subjects evaluated the three test solutions and performed a sensory evaluation. The salt concentration of soup consumed by Japanese individuals is generally 0.9%. Thus, the three different concentrations of stock soups were tasted in order from A to C in a double-blind manner (commercially available soup with 0.9% NaCl solutions (A), commercially available soup with 0.6% NaCl solutions (B), and reborn soup diluted to 0.6% NaCl solutions (C)). Ingredients of the commercially available soup were dried bonito and soy sauce (the equivalent amount of salt was 17.8 g/100 g), and the reborn soup was made from chicken bones, onion, ginger, garlic, shiitake mushrooms, and kelp (the equivalent amount of salt was 0.1 mg/100  g), which were provided by Reborn TK Japan Co. Ltd. (Fukuoka, Japan). A was made to the basic concentration of 0.9%, and B was made to a concentration of 0.6% by adding water. We diluted the reborn soup to make the salt concentration 0.6%. All soups were kept warm at 80–90°C and then poured into paper cups, and served at 65–70°C for sensory evaluation. Subjects were asked to rinse their mouth with pure water before tasting and to spit out the solutions after holding them in their mouths. Evaluations of saltiness and palatability for each solution were conducted using a visual analog scale (VAS) in a double-blinded randomized manner (Table 1).

We estimated 24-hour salt excretion by measuring sodium (Na) and creatinine (Cr) concentrations and using a calculation formula. This method is practical at general medical facilities and is also a recommended method for evaluating typical daily salt intake by the Working Group for the Dietary Salt Reduction of the Japanese Society of Hypertension [15]. The reliability of estimated 24-hour salt excretion has been improved by using a calculation formula that incorporates the estimated 24-h urinary Cr excretion based on age, height, and body weight; the formula is as follows: estimated salt intake (g/day) = {21.98 × (Na concentration in a spot urine sample (mEq/l)/Cr concentration in a spot urine sample (mEq/l)) × (−2.04 × age + 14.89 × body weight (kg) + 16.14 × height (cm) − 2244.45)}0.392 × 0.0585. Fasting urine samples during the morning checkup were collected and estimated salt intake was assessed using this formula. Participant's estimated salt intake levels were categorized into tertiles (Q1: <7.95 g/day; Q2: 7.95–9.98 g/day; Q3: ≥9.99 g/day).

Clinic BP was measured with an automatic sphygmomanometer while the subject was seated. Hypertension was considered to be present in patients with systolic BP of ≥ 140 mmHg and/or diastolic BP of ≥ 90 mmHg, or the use of antihypertensive medication. The goal BP was defined as <140/90 mmHg.

In addition, we also assessed medication, other comorbidities, and awareness of salt intake using a self-description questionnaire about lifestyle items such as meal content and amount, frequency of dining out, and use of seasoning among the study subjects (Table 2). Each item was scored (total score: from 0 to 35), with a low score reflecting an awareness of the desirability of low salt intake. This sheet showed a significant positive correlation between the total score and estimated 24-hour salt excretion, making it possible to easily evaluate salt intake trends [16].

The protocol was explained in detail and informed consent was obtained from each subject. This study was performed in accordance with institutional guidelines and approved by the institutional ethical committee (no. 22-1).

### 2.1. Statistical Analysis

Values are presented as mean ± standard deviations (SDs). Student's *t*-test and a chi-square test were used when appropriate. All calculations were performed using a standard statistical package (JMP 10; SAS Institute, Cary, NC, USA). *P* values <0.05 were considered significant.

## 3. Results

**Figure 1:**
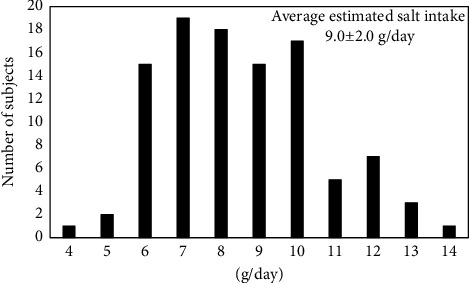
Estimated salt intake of all patients (*N* = 103).

**Figure 2:**
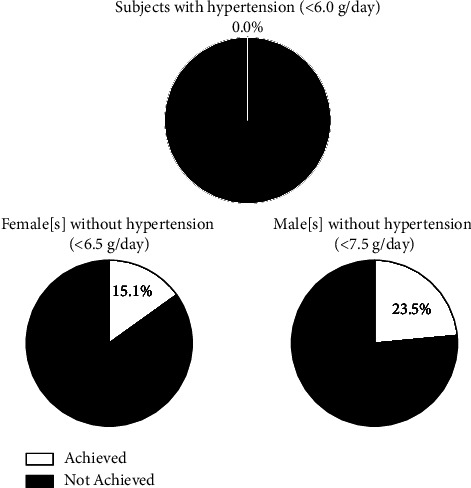
Rate at which established salt intake goal was being met for each goal category.

**Figure 3:**
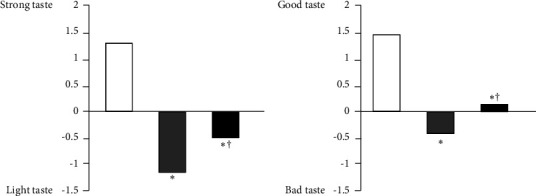
Evaluations of saltiness (a) and palatability (b) for each soup (*N* = 103); white bars: commercially available soup with 0.9% NaCl solutions, gray bars: commercially available soup with 0.6% NaCl solutions, and black bars: reborn soup diluted to 0.6% NaCl solutions. ^∗^*p* < 0.01 vs. 0.9% NaCl solutions. ^†^*p* < 0.01 vs. 0.6% NaCl solutions.

**Figure 4:**
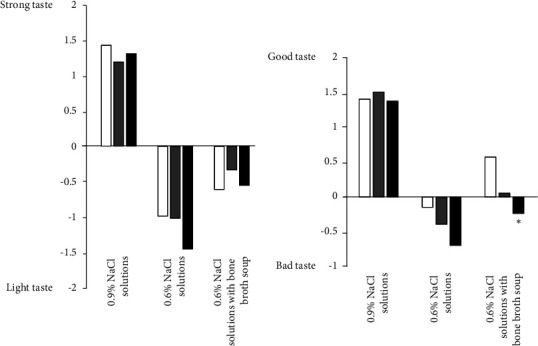
Evaluations of saltiness (a) and palatability (b) for each soup; white bars: estimated salt intake <7.95 g/day, gray bars: estimated salt intake 7.95–9.98 g/day, and black bars: estimated salt intake ≥9.99 g/day. ^∗^*p* < 0.05 vs. NaCl <7.95 g/day.

The characteristics of our subjects are shown in Table 3. The mean age was 35 ± 12 years, and 21% of the subjects were male. The average clinic BP was 118 ± 13/75 ± 10 mmHg in all subjects. Among our subjects, 13% were hypertensive.  Figure 1 shows the estimated salt intake in all subjects. Results were widely distributed with an average estimated salt intake of 9.0 ± 2.0 g/day. JSH 2019 proposed a salt reduction goal of <6 g/day for hypertensives, and DRI-J 2020 set daily salt intake targets as <7.5 g/day and <6.5 g/day for nonhypertensive men and women, respectively [3, 4]. The achievement rates of the estimated salt intake in 73 females without hypertension (<6.5 g/day), 17 males without hypertension (<7.5 g/day), and 13 participants with hypertension (<6.0 g/day) were 15.1%, 23.5%, and 0.0%, respectively (Figure 2). In the taste tests, B was rated significantly lower than A for both saltiness and palatability, but C was rated significantly higher than B (Figure 3). Participant's estimated salt intake levels were categorized into tertiles (Q1: lowest; Q3: highest). There was no difference in the perception of saltiness in C between the Q1 and Q3 groups (−0.6 ± 1.3 vs −0.6 ± 1.4, ns, Figure 4(a)); however, the palatability of C was significantly higher in the Q1 group than in the Q3 group (0.6 ± 1.4 vs −0.2 ± 1.1, *p* < 0.01, Figure 4(b)). The urinary sodium-to-potassium ratio and the total average score for the awareness of salt intake on the questionnaire in the Q1 group was significantly lower compared to that in the Q3 group (2.3 ± 2.3 vs 4.5 ± 2.2, *p* < 0.01; 11.6 ± 5.1 vs 13.5 ± 4.5, *p* < 0.05, Table 4).

## 4. Discussion

The results of our analyses were twofold. Most subjects were not meeting the established goals for salt intake, and those who were aware of the desirability of low salt intake had a lower estimated salt intake. In the sensory assessment, the effect of reborn soup led to increased palatability and a lesser degree, perception of saltiness, when compared with a solution containing the same amount of salt but no reborn soup. This was especially true for the group with the lowest salt intake.

The JSH 2019 adopted the salt reduction goal of <6 g/day [3], while the European Society of Cardiology (ESC)/European Society of Hypertension (ESH) Treatment Guidelines 2018 proposed an even stricter goal of <5 g/day [17]. Regarding the goal of a reduction in salt intake for the general population, DRI-J 2020 proposed the goal of salt intake of <7.5 g/day for men and <6.5 g/day for women [4]. On the other hand, the WHO guidelines for the general population strongly recommended a salt intake of <5 g/day [18]. Although the average salt intake gradually has decreased during the past 10 years, the latest survey results (2019) showed that the average salt intake was 10.9 g/day for men and 9.3 g/day for women. We previously investigated the effectiveness of repeated assessment of 24 h urinary salt excretion with the guidance of salt reduction provided by either doctors or dieticians in hypertensive outpatients [6]. Over 8.6 years, average urinary salt excretion showed a significant decrease from 9.6 g/day to 8.2 g/day; however, it was difficult for subjects to achieve the target level of salt intake (<6 g/day). Other approaches including the utilization of low-salt foods are necessary to achieve further reduction of salt intake, especially in Japanese consumers, who are accustomed to higher-salt cuisines. It is especially important to find ways of preparing salt-reduced foods that the Japanese find palatable, and the additions of MSG, CDG, and herbs have been well-known strategies [9–14, 19]. Reborn soup was made from chicken bones, onion, ginger, garlic, shiitake mushrooms, and kelp. Onion, ginger, garlic, and kelp contain glutamic acid, and shiitake mushrooms contain guanylic acid. Chicken bones contain all three umami components (glutamic acid, inosinic acid and guanylic acid). These are known as umami substances, and it has been proven that combining these ingredients enhances the umami taste [20]. It is not clear why reborn soup was more effective than B soup; however, the synergistic effects of various umami components may to be involved. Although it should be considered that the complexity of taste perception in the brain may affect sensory evaluation, our study indicated that reborn soup may have the capability to compensate for the loss of palatability caused by salt reduction.

There was no difference in the evaluation of the saltiness of reborn soup between the low- and high-salt-intake groups; however, the low-salt-intake group evaluated the palatability of reborn soup significantly higher than the high-salt-intake group. It is known that saltiness sensitivity decreases with aging [21], but there was no difference in age between the low- and high-salt-intake groups (34 ± 11 years vs 35 ± 11 years, ns). It was previously reported that 45.3%, 28.0%, and 18.7% of subjects with diabetes showed lower umami taste sensitivity, lower sweet taste sensitivity, and lower salt taste sensitivity, respectively [22]; however, there was no difference in the frequency of diabetes between the low- and high-salt-intake groups (0.0% vs 2.9%, ns). Smoking was also reported to cause an increase in the salt taste threshold [23], but there was no difference in frequency of smoking between the low- and high-salt-intake groups (5.9% vs 17.6%, ns). Interestingly, it was reported that salt-loaded mice showed no change in preference or sensitivity to sour taste, but decreased preference/sensitivity to sweet, umami, and bitter tastes [24]. Thus, high-salt-intake individuals may either inherently have or develop taste disorders. In addition, it was reported that taste preferences established under the influence of food experiences in early childhood continue through life [25]. The best estimate of early taste experiences is the region of birth; however, we did not assess the level of salt intake by regional block at birth in our subjects.

It is challenging for the food industry to reduce sodium in foods without fully understanding the impact of sodium reduction on the sensory properties of foods. Our result may provide guidance on preparing palatable salt-reduced foods. The utilization of reborn soup appeared to be useful in enhancing salty taste in both hypertensive patients as well as in normotensive subjects; apart from its inherent nutritional value, its use would be beneficial for reducing salt content in food, and thus positively impact cardiovascular disease and mortality. Our findings could also be used to invent new seasonings to improve the palatability of salt-reduced food.

The present study has several limitations. First, our subjects were medical staff and there were sex and age biases. Our subjects seemed to prefer less salt intake than the general population because of high health awareness; however, the rates at which their estimated salt intake met established targets were low. Second, we estimated salt intake using spot urine samples in the morning. Estimated salt intake may change according to the urine sample time because urinary salt excretion has a circadian rhythm and may also be influenced by the time at which the food is consumed. In addition, a single measurement may not be sufficient to assess the salt intake of individuals because salt intake is known to change daily and appears to also be influenced by changes in dietary habits. However, spot urine samples are practical at general medical facilities, and the reliability of the findings obtained may be improved using a calculation formula incorporating the estimated 24-h urinary Cr excretion based on age, height, and body weight [15]. Third, saltiness and palatability were assessed by a VAS in the present study. The VAS may have problems with reproducibility, individual differences, and objectivity; however, it is usually used as an appropriate method for evaluating the sensation induced by tastes and is confirmed to have reproducibility and validity in assessment [26]. Fourth, the flavors were different between commercially available soup (seafood-based) and reborn soup (meat-based). We used seafood-based commercially available soup as our control soup because it was the most used in Japan. Another limitation is that the present investigation was conducted in one institution and the sample size was small. Therefore, the results obtained may not be representative of the current status of the general Japanese population. Further large-scale studies seem to be needed to confirm the sensory effect of reborn soup in enhancing perceptions of saltiness and palatability.

In conclusion, most randomly sampled subjects were not meeting the established goals of salt intake. The high-salt-intake group might be less able to respond to other flavors, including umami, that make foods palatable. Our results indicate that reborn soup would likely be effective in improving the palatability of reduced-salt foods.

## Figures and Tables

**Table 1:** Sensory evaluations: saltiness and palatability.

Saltiness	Light taste −3	−2	−1	Normal taste 0	+1	+2	Strong taste +3
Palatability	Bad taste −3	−2	−1	Normal taste 0	+1	+2	Good taste +3

**Table 2:** Self-description questionnaire on the awareness of salt intake.

	Score	3	2	1	0
Frequency	Soup	2 times/day≤	1/day	2-3 times/week	Never
	Salt pickles and pickled plum	2 times/day≤	1/day	2-3 times/week	Never
	Boiled fish paste	—	Often	2-3 times/week	Never
	Salted salmon	—	Often	2-3 times/week	Never
	Ham and sausage	—	Often	2-3 times/week	Never
	Noodles	Every day	2-3 times/week	≤1/week	Never
	Rice crackers and chips	—	Often	2-3 times/week	Never
	Seasoning	Often (each meal)	1/day	Sometimes	Never

Do you drink the juices of noodles?		All	Half	A little	Never

Do you dine out or go to convenience stores at lunch?		Every day	3 times/week	1/week	Never

Do you dine out or cook for dinner?		Every day	3 times/week	1/week	Never

How does the food you cook yourself compare to dining out?		Rich taste	Similar	—	Bland taste

What size portions do you eat?		Large	—	Average	Small

Total score: from 0 to 35. (0–8, low salt intake; 9–13, average salt intake; ≥14, high salt intake).

**Table 3:** Characteristics of subjects (*N* = 103).

Age (year)	35 ± 12
Male (%)	21
Current smoker (%)	9
Body mass index (kg/m^2^)	21.1 ± 3.2
Hypertension (%)	13
Blood pressure (mmHg)	118 ± 13/75 ± 10
Estimated salt intake (g/day)	9.0 ± 2.0
Urinary sodium/potassium ratio	3.3 ± 2.2
Average score for the awareness of salt intake	12.1 ± 4.5

All values are given as the mean ± SD or percentage.

**Table 4:** Characteristics of subjects on salt intake level.

Estimated salt intake (g/day)	<7.95 (*N* = 34)	7.95–9.98 (*N* = 35)	≥9.99 (*N* = 34)
Age (year)	34 ± 11	36 ± 14	35 ± 13
Male (%)	18	23	24
Current smoker (%)	6	3	18
Body mass index (kg/m^2^)	20.7 ± 2.9	20.4 ± 2.6	22.2 ± 3.7^#^
Hypertension (%)	6	11	21
Blood pressure (mmHg)	117 ± 12/73 ± 8	116 ± 11/74 ± 9	120 ± 15/78 ± 12
Estimated salt intake (g/day)	6.8 ± 0.8	8.8 ± 0.6^∗∗^	11.4 ± 1.2^∗∗^^##^
Urinary sodium/potassium	2.3 ± 2.3	3.0 ± 1.2	4.5 ± 2.2^∗∗^^##^
Average score for the awareness of salt intake	11.6 ± 5.1	11.3 ± 3.7	13.5 ± 4.5^#^

All values are given as the mean ± SD or percentage. ^∗∗^*p* < 0.01 vs. <7.95 g/day. ^##^*p* < 0.01. ^#^*p* < 0.05 vs. 7.95–9.98 g/day.

## Data Availability

The data used to support the findings of this study are available from the corresponding author upon request.
